# Lack of Fetal Protection against Bovine Viral Diarrhea Virus in a Vaccinated Heifer

**DOI:** 10.3390/v14020311

**Published:** 2022-02-02

**Authors:** Małgorzata D. Klimowicz-Bodys, Mirosław P. Polak, Katarzyna Płoneczka-Janeczko, Emilia Bagnicka, Dominika Zbroja, Krzysztof Rypuła

**Affiliations:** 1 Division of Infectious Diseases of Animals and Veterinary Administration, Department of Epizootiology and Clinic of Birds and Exotic Animals, Faculty of Veterinary Medicine, Wroclaw University of Environmental and Life Sciences, pl. Grunwaldzki 45, 50-366 Wroclaw, Poland; malgorzata.klimowicz-bodys@upwr.edu.pl (M.D.K.-B.); katarzyna.ploneczka-janeczko@upwr.edu.pl (K.P.-J.); 2 Department of Virology, National Veterinary Research Institute, al. Partyzantów 57, 24-100 Pulawy, Poland; ppolak@piwet.pulawy.pl; 3 Department of Biotechnology and Nutrigenomics, Institute of Genetics and Animal Biotechnology Polish Academy of Sciences, ul. Postepu 36A, Jastrzębiec, 05-552 Magdalenka, Poland; e.bagnicka@igbzpan.pl; 4 Student Scientific Society “AnthraX”, Department of Epizootiology and Clinic of Birds and Exotic Animals, Faculty of Veterinary Medicine, Wroclaw University of Environmental and Life Sciences, pl. Grunwaldzki 45, 50-366 Wroclaw, Poland; 113196@student.upwr.edu.pl

**Keywords:** BVDV, persistently infected, vaccination

## Abstract

The aim of the report was to present the circulation of BVDV (bovine viral diarrhea virus) in the cattle population and determine the cause of the failure of vaccination failure leading to the birth of the PI (persistently infected) calf. The case study was carried out at the BVDV-free animal breeding center and cattle farm, where the vaccination program against BVDV was implemented in 2012, and each newly introduced animal was serologically and virologically tested for BVDV. In this case, a blood sample was taken from a 9-month-old breeding bull. Positive RT-PCR and negative ELISA serology results were obtained. The tests were repeated at 2-week intervals, and the results confirmed the presence of the virus and the absence of specific antibodies, i.e., persistent infection. Additionally, sequencing and phylogenetic analysis were performed, and the BVDV-1d subgenotype was detected. The results of this study showed that pregnant heifers and cows that are vaccinated multiple times with the killed vaccine containing BVDV-1a may not be fully protected against infection with other subgenotypes of BVDV, including their fetuses, which can become PI calves.

## 1. Introduction

The bovine viral diarrhea virus (BVDV) is a threat to cattle health and causes significant economic losses for cattle farming. Because of these losses, bovine viral diarrhea (BVD) is considered one of the most important infectious diseases of cattle [1,2,3]. According to the adopted nomenclature, Pestivirus A and Pestivirus B (formerly BVDV-1 and BVDV-2, respectively) [4] are heterogeneous viruses belonging to the genus *Pestivirus* and the family *Flaviviridae* that differ from each other in terms of antigenic and virulence properties. At least 23 Pestivirus A subgenotypes, known as BVDV-1a–1w, and four Pestivirus B subgenotypes, known as BVDV-2a–2d, have been identified [5,6,7]. The largest number of subgenotypes was reported in European countries, indicating the possibility of greater genetic diversity of the virus in Europe compared with that of other continents. In Europe, the most frequent subgenotypes are -1a, -1b, -1d, -1e, -1f, and -1h [6].

BVDV can be transmitted horizontally, leading to transient infection (TI); however, an essential factor in the epidemiology of BVDV is the ability of the virus to infect the fetus [8]. When infected early in pregnancy and before the immune system develops, that is, generally between days 42–125 of pregnancy [9], the virus can cause persistent infection, resulting in the birth of a persistently infected calf (PI) [10]. The fetal immune system recognizes the infecting virus as self and is unable to mount a strong innate and adaptive immune response to the virus. A persistently infected calf continuously sheds virus in large quantities [11] in all secretions and is the most important source of infection for susceptible cattle [12]. Therefore, persistently infected animals are a major target of compulsory or voluntary disease control and eradication programs that have been implemented in many countries [13,14,15,16]. However, there is no universal approach to the design of a specific BVDV eradication program because of the variability of important factors that influence viral spread, such as the density of susceptible animals, their movement, and the prevalence of BVDV [13,14].

The first vaccines against BVDV were planned to provide protection from clinical disease, but later on, when the pathogenesis of BVDV infection and especially the creation of PI animals were elucidated, it became crucial for vaccines to offer fetal protection in pregnant females. In a meta-analysis of previous studies, Newcomer et al. [17] evaluated the influence of vaccines on reproductive losses, such as fetal infection or abortion. It was concluded that any vaccination provided significant protection against reproductive disease and fetal infection, which could be reduced by up to 85%. However, the well-known genetic diversity of RNA viruses, including BVDV, can have a negative impact on successful vaccination. Surprisingly, 100% protection against PI calf birth was achieved after vaccination with an inactivated vaccine, but only when the vaccine and field strain of BVDV were of the same subgenotype [18]. In a recent study from Poland, in five vaccinated herds, 17 PI animals were identified as being infected with two BVDV subgenotypes that were different from the vaccine strain [19]. The aim of the study was to identify the possible reason for the birth of a PI calf in a vaccinated heifer.

## 2. Materials and Methods

### 2.1. Animal and Samples Collection

Nineteen 9-month-old breeding bulls were tested for bovine viral diarrhea (BVD) (serology and virology) in March 2021. The animals were born and kept at the Animal Breeding Center (Farm A) in the southwestern part of Poland. Blood samples were collected from the jugular vein by a field veterinarian who worked on the farm. Blood was left at room temperature for 8–12 h after collection and then centrifuged. Serum samples were then frozen at −80 °C and transported directly to the Diagnostic Laboratory of the Department of Virology of the National Veterinary Research Institute, Pulawy, Poland, for the detection of viral RNA and antibodies using RT-PCR and ELISA, respectively. Additionally, DNA sequencing and phylogenetic analysis were performed to identify the viral subgenotype.

### 2.2. Detection of Viral RNA

Total RNA was extracted from 500 µL of serum using TRI Reagent (Sigma–Aldrich, Burlington, MA, USA), according to the manufacturer’s protocol. Reverse transcription-polymerase chain reaction (RT-PCR) was performed with Titan One Tube RT-PCR System kit reagents (Roche, Germany) following the manufacturer’s instructions. The sequences of primers used were the following: 324F (5′-ATG CCC WTA GTA GGA CTA GCA-3′) and 326R (50-TCA ACT CCA TGT GCC ATG TAC-30) [20]. The size of the PCR product for the 5′UTR region was estimated at 288 bp. The RT-PCR reaction mixture of 25 µL was made of 14 µL of RNase-free water, 5 µL of reaction buffer, 0.5 µL of the dNTP mixture (10 mM), 1 µL of each primer (10 mM), 1.25 µL of the DTT solution (100 mM), 0.5 µL of enzyme mix, and 2 µL of extracted RNA. Amplification was performed with 35 cycles consisting of denaturation at 94 °C for 15 s, annealing at 53 °C for 30 s, and extension at 68 °C for 30 s.

### 2.3. Antibody Detection

Serum samples were tested with Erns Ab ELISA (BVDV Total Ab Test, IDEXX, Liebefeld-Bern, Switzerland). The ELISA test was performed according to the manufacturer’s instructions, and samples with S/P values higher than or equal to 0.3 were classified as positive. The test provides a specificity and sensitivity of 97.1% and 96.7%, respectively, compared with the virus neutralization test (VNT) [21].

### 2.4. DNA Sequencing and Phylogenetic Analysis

The 5′UTR amplicon was gel-purified and sequenced in both directions using the same primers as those for RT-PCR with the Big Dye Terminator v3.1 Cycle Sequencing Kit and the 3730XL Genetic Analyzer (Applied Biosystems, Foster City, CA, USA). The fragment of DNA was purified with the QIAquick PCR Purification Kit (Qiagen, Hilden, Germany) and then analyzed on a 16-capillary sequencer ABI PRISM 3100 Genetic Analyzer (Applied Biosystems). The results were analyzed using the GeneScan Analysis Software (Applied Biosystems). Sequences were generated, and the consensus sequence was prepared with the CAP3 Sequence Assembly Program [22]. CLUSTALW software [23] was used for sequence alignment. MEGA software (version 5.05) was used for phylogenetic analysis, while bootstrap analysis was performed on 1000 replicates. The phylogenetic tree was constructed using the neighbor-joining and maximum likelihood methods with 1000 bootstrap replicates [24].

## 3. Results

### 3.1. Test Results

**Figure 1:**
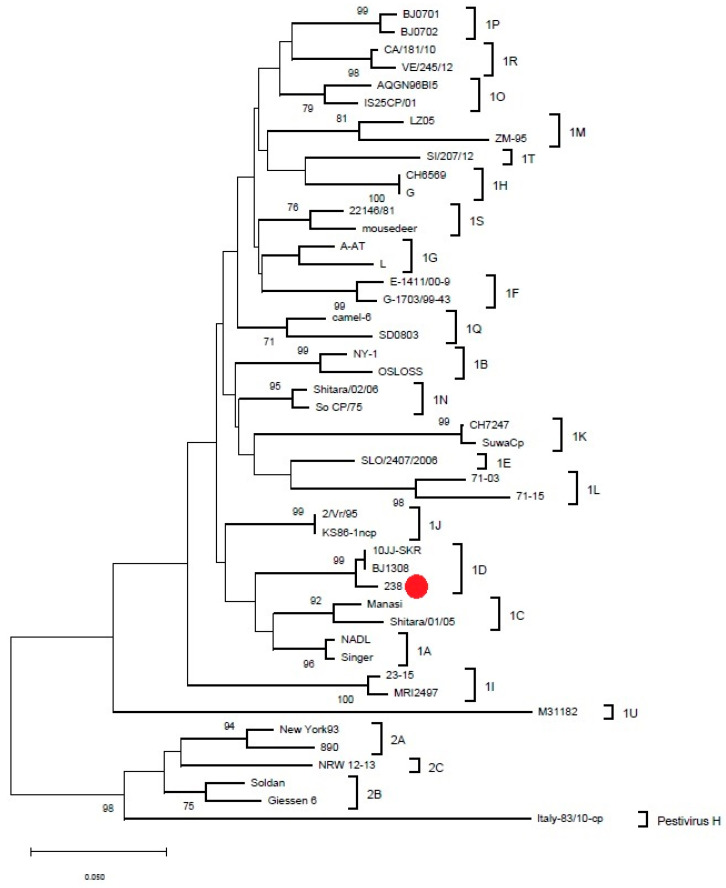
The phylogenetic tree. The red dot indicates the BVDV-1d subgenotype of the male calf in this study (no. 238). BVDV sequences used to generate phylogenetic tree with GenBank accession numbers are provided as they appear in the figure from top to bottom: sequence ID (GenBank number): BJ0701 (GU120247.1), BJ0702 (GU120248.1), CA/181/10 (LM994672.1), VE/245/12 (LM994671.1), AQGN96BI5 (AB300691.1), IS25CP/01 (AB359931.1), LZ05 (GU120241.1), ZM-95 (AF526381.3), SI/207/12 (LM994674.1), CH6569 (MH907191.1), G (AF298066.1), 22146/81 (AJ304386.1), Mousedeer (AY158154.1), A-AT (FJ493482.1), L (AF298069.1), E-1411/00-9 (AY323872.1), G-1703/99-43 (AY323876.1), camel-6 (KC695810.1), SD0803 (JN248727.1), NY-1 (FJ387232.1), OSLOSS (AY279528.1), Shitara/02/06 (LC089876.1), So CP/75 (AB042661.1), CH7247 (MH907869.1), SuwaCp (AF117699.1), SLO/2407/2006 (KX577637.1), 71-03 (KF205294.1), 71-15 (KF205306.1), 2/Vr/95 (AJ293594.1), KS86-1ncp (AB078950.1), 10JJ-SKR (KC757383.1), BJ1308 (KF925517.1), 238 (the isolate from this study—The sequence of this isolate was submitted to GenBank under provisional accession number OM142638), Manasi (EU159702.1), Shitara/01/05 (AB359926.1), NADL (AJ133738.1), Singer (L32875.1), 23-15 (AF298059.1), MRI2497 (LT902628.1), M31182 (JQ799141.1), New York93 (KR093034.1), 890 (L32886.1), NRW 12-13 (HG426483.1), Soldan (AY735495.1), Giessen 6 (AY379547.1), Italy-83/10-cp (JQ612705.1).

For one breeding bull, a positive test result for the presence of viral nucleic acid (RT-PCR) was obtained; however, it was negative for the presence of specific antibodies (ELISA). The tests were repeated twice, 2 weeks apart, and in both tests, the results confirmed the presence of viral genetic material and the absence of antibodies. The bull did not seroconvert after vaccination, unlike the rest of the tested animals that were serologically positive. In the remaining animals, the test results for the presence of viral RNA were negative. Sequencing and phylogenetic analysis were performed on this particular animal only, and the result is presented in Figure 1.

### 3.2. Epizootic Investigation

#### 3.2.1. Vaccination against BVD at the Animal Breeding Center (Farm A)

At the Animal Breeding Center (Farm A), a vaccination program against BVD was introduced in 2012. The rationale for introducing vaccination was the presence of two persistently infected heifers born from antigen-negative cows. They were removed from the herd, and it was decided that all newborn animals would be tested for the presence of BVDV genetic material. Then the vaccination program for all animals was started and carried out in the following years in a herd in which there was no reservoir for BVDV. The killed vaccine Bovilis^®^ BVD containing the inactivated antigen of the cytopathic BVD virus strain C-86 subgenotype 1a (Intervet International BV, The Netherlands) was used as follows: first vaccination at 6 months of age, repeated after 4 weeks, and sequential vaccination every 6 months.

#### 3.2.2. History of the Bull and His Dam

The mother of the bull was purchased as a 9-month-old heifer from the BVDV and bovine herpesvirus type 1 (BHV-1)-free herd in the Netherlands in May 2019. The health certificate showed that this heifer had been tested for BVDV antigen and antibodies in the Netherlands. The results of these tests were negative. Upon arrival at Farm A, an RT-PCR test was performed to detect the presence of BVDV genetic material. The result of this test was also negative.

After receiving the test results, the heifer was vaccinated against BVD at 10 and 11 months of age (June and July 2019) with the killed vaccine Bovilis^®^ BVD (Intervet International BV, Boxmeer, The Netherlands) according to the vaccination program, introduced at Farm A. At the same time, all animals were vaccinated against infectious bovine rhinotracheitis/infectious pustular vulvovaginitis (IBR/IPV) with the Rispoval IBR-Marker Inaktivatum^®^ vaccine (Zoetis Belgium SA, Louvain-la-Neuve, Belgium).

In late July, the vaccinated heifer was transported to an insemination center (Farm B) in the northeast part of Poland. However, only those animals that had negative test results for the presence of BVDV genetic material and antibodies were introduced to Farm B. All animals on this farm were vaccinated against BVD using the killed vaccine, so 4 weeks after arriving at the farm, the heifer was vaccinated. In total, the heifer was vaccinated three times against BVD within 2 months. The described heifer spent about 5 months on Farm B, served as a donor for embryo transfer, and was subjected to systematic stress due to regular non-surgical embryo collection procedures. Semen for artificial insemination was examined and was free from BVDV. In October 2019, the heifer was inseminated for the last time, and after 7 weeks, it was transported to Farm A.

After returning, the 19-month-old heifer was vaccinated with the modified live vaccine (MLV) Mucosiffa^®^ containing cytopathic BVDV-1a strain Oregon C24 strain (CEVA Animal Health, Liburne, France) in March 2020, following the manufacturer’s instructions.

**Figure 2:**
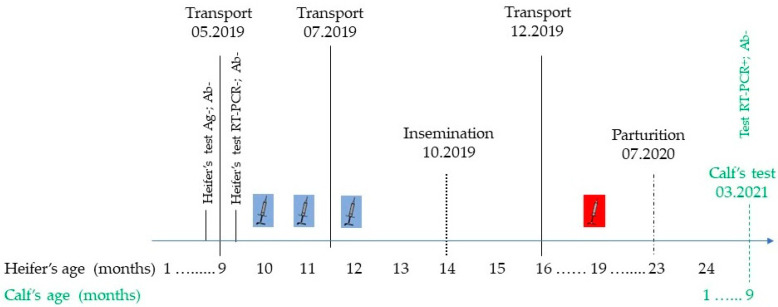
Chronology of the events. 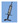 killed vaccine; 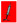 MLV vaccine.

In July 2020, the heifer gave birth to a male calf. After birth, the offspring were housed separately, with individual access to feed and water. The calf was tested at the age of 9 months prior to planned transport to Farm B. The results obtained are described above. Because of safety reasons, the PI animal was euthanized. The chronology of the events is presented in Figure 2.

## 4. Discussion

Identifying the possible time of infection with BVDV is complicated, and in many cases, it is not possible to determine the exact time at which it happened. This is also the case for this study, in which we identified a PI bull from a vaccinated heifer without any clear evidence of when transplacental infection could have occurred. The mother of the infected bull was transported from one farm to another and back to the first one, and its health status was not monitored during these activities (i.e., by laboratory testing), so we can only suspect the most probable time of infection. It is well known that the generation of PI animals is possible in the early stages of gestation (usually between days 42–125) [9], although it can also take place earlier, usually at least 30 days after insemination [25]. The heifer in question was kept for 49 days after insemination on Farm B and then transported back to Farm A. Therefore, the generation of PI could have taken place on Farm B from 30 days after insemination, during transport to Farm A, or on Farm A. At the time of the study, both farms applied strict biosafety measures to avoid the introduction of PI animals by testing all new arrivals and vaccinating all animals in these herds after the removal of PI. Therefore, we assumed that it was highly unlikely that the infection took place on Farm A or B. In our opinion, the critical period could have been day 49 of gestation, when the pregnant heifer was transported back to Farm A. We do not have any evidence on the health status of other ruminants transported with this heifer, and we can suspect that at least one of them could be infected with BVDV, even transiently. It is well known that one hour of direct contact of the susceptible animal with the infected individual is enough to pass the infection. In this case, the transport took up to 7 h.

Animal movement is one of the risk factors for virus spread. This environment, in which infected and susceptible animals share the same limited space during transport, and high concentrations favor the transmission of the virus among the animals. Transport-associated stress can alter immune functions, potentially compromising the animal’s welfare by increasing the likelihood of infection and/or clinical disease. Among transport-related health problems, BVDV infections are common and can be severe [26,27,28]. A widespread route of spreading the virus is possible through the movement of PI cattle between herds [29,30], which is highly probable in herds in which the movement frequency is high [31]. Another possibility is that the virus could be transmitted through people, machines, or means of transport. The washing and disinfection of the means of transport should be carried out after each movement of the animals. During the investigation, no information was available to prove whether such activities had been performed or whether the cleanliness of the lorry was checked before transport. Therefore, the authors could not exclude a situation in which the lack of proper washing and disinfection was the cause of the heifer’s infection during transport.

The constant stress to which the heifer was exposed (transport, repeated stimulation of superovulation, insemination, and embryo collections) could be the cause of immunosuppression. Many hypotheses about the relationship between transport and infection and the appearance of a bovine respiratory disease complex (including BVD) are known [32]. Many researchers agree that the numerous stressors that animals experience during transport can cause a general decrease in immunity and can allow numerous pathogens to invade the respiratory system [27]. Transport creates stressful conditions that can increase susceptibility to infection, although in the case of BVD, it is not as important as in the case of BHV-1, in which latent infection can be reactivated [33]. 

To evaluate the impact of the stress factors mentioned above, including transport, “stress” assessment trials, such as measuring cortisol levels and assessing immune function, should be performed. However, blood samples from the transport period for this study were not available.

One of the tools for controlling and eradicating BVD is vaccination. Many authors indicate that vaccination with inactivated vaccines does not provide satisfactory protection against fetal infection. However, a study by Patel et al. [18] showed that the vaccine can provide complete protection to the bovine fetus although only against homologous species of BVDV. The authors observed that calves vaccinated with the killed vaccine based on BVDV-1a could become infected with BVDV-1b after challenges with PI calves. Other authors also showed that two doses of inactivated vaccine containing different subgenotypes of BVD virus administered to heifers during the 69 days prior to exposure did not provide high protection, and 27% of calves were persistently infected [34]. Rodning et al. [35] recorded similar observations on the effectiveness of vaccines comparing two commercially available multivalent vaccines containing modified-live BVDV (containing CP, type 1a, and type 2 BVDV) and one commercially available multivalent vaccine containing inactivated BVDV (containing CP type 1a, NCP type 1a, and NCP type 2 BVDV). Four doses of inactivated vaccine administered from weaning to insemination provided 89% protection against fetal development, while four doses of the MLV vaccine provided 100% protection against the generation of PI calves.

In the present study, the heifer was vaccinated three times with an inactivated vaccine for several months before the final insemination. Therefore, in theory, up to about day 125 of gestation, there was potential protection against infection, which should result in a low risk of the generation of a PI fetus in this animal. Before 125 days of gestation, infection of the fetus with noncytopathic BVDV usually results in the generation of a PI fetus. Noncytopathic BVDV is the only biotype that has been observed clinically or experimentally to cause persistent infection with BVDV [25,36]. Therefore, the vaccination of a pregnant heifer from this study with a modified live vaccine containing a cytopathic strain of BVDV should not be regarded as a possible reason for the generation of a PI fetus generation. Furthermore, the heifer was vaccinated in the fifth month of gestation, preventing persistent infection of the already immunocompetent fetus. 

In the authors’ opinion, another aspect—the phylogenetic variability of BVDV strains—should be considered. The largest number of subgenotypes (at least 16: 1a–1l and 1r–1u) was reported in European countries, indicating a greater genetic diversity of the virus in Europe compared with other continents. The most frequently reported BVDV-1 subgenotypes are 1a and 1b; 1e, 1f, 1h, and 1d are less frequently reported [6]. In the years 2004–2011, a study using phylogenetic analysis (5’UTR sequences and full-length N^pro^ encoding sequences of BVDV-1) was carried out in Poland, in which the presence of four BVDV-1 subgenotypes and one BVDV-2 (a) was confirmed. The dominant BVDV subgenotypes were BVDV-1b and -1d, and the less frequent subgenotypes were 1f and 1g, while 1a was not found [37]. A recent study from 2015–2018 identified seven BVDV subgenotypes in Poland in descending order of frequency of appearance: 1b, 1g, 1f, 1d, 1r, 1s, and 1e; however, BVDV-1b was still the most frequently detected subgenotype [38].

Patel et al. [18] found that despite the use of vaccines containing BVDV-1a and BVDV-2 in mothers, their PI offspring were infected with the BVDV-1b subgenotype. Studies carried out in Poland showed that multiple vaccinations of cows with inactivated vaccines containing BVDV-1a did not protect fetuses against infection with a genetically separate strain of BVDV, despite the high antibody titer found in mothers [19]. Other authors [39] have shown that MLV vaccines containing BVDV-1a strains induce fewer antibodies against BVDV-1b than against BVDV-1a and thus probably do not provide complete protection against BVDV-1b infection. Lower titers of BVDV-1b antibodies could potentially indicate reduced animal protection when exposed to heterologous BVDV-1b. Sozzi et al. [40], using four commercial vaccines (three MLV containing BVDV-1a or BVDV-1b and one inactivated containing BVDV-1a), showed, among other findings, that the virus-neutralizing antibody response induced by BVDV vaccines was completely absent against BVDV-1e, thus indicating the existence of significantly different epitopes effective for neutralization between BVDV-1e and the other subgenotypes, such as BVDV-1a and BVDV-1b.

Therefore, it should be emphasized that different levels of cross-protection between highly variable BVDV-1 subgenotypes may influence the success of vaccination programs [6]. One cannot exclude the possibility of low antibody titers due to vaccination, which do not provide efficient protection of the dam (and eventually of the fetus) from infection with the field strain of BVDV. Therefore, two explanations could be given. One is that the viral subgenotype in the vaccine used was different from the field strain, which did not protect the female from infection (and eventually also the fetus), and the other is that the antibody titer was too low to protect both the pregnant heifer and its progeny despite vaccination. Several studies indicated that the VN titer after vaccination should be high enough (at least 128) to protect the fetus of the vaccinated dam from intrauterine infection [41]. Since we did not perform VN tests, we could not provide this information.

In conclusion, vaccination cannot be considered the optimal method to protect animals and developing fetuses from BVDV infection, especially when the viral subgenotype present in the vaccine differs from the field isolate, which can compromise the antibody response and lead to the birth of PI calves. Other factors, such as transport and stress-related procedures (embryo transfer), should also be considered when evaluating the possible risk of BVDV introduction to farms.

## Data Availability

The datasets used and/or analyses during the current study are available via e-mail from the corresponding author on reasonable request. The sequence of BVDV-1d isolate of the male calf in this study was submitted to GenBank under provisional accession number OM142638.
